# EUS and ERCP training in Europe: Time for simulation, optimization, and standardization

**DOI:** 10.1002/ueg2.12399

**Published:** 2023-05-07

**Authors:** Selma J. Lekkerkerker, Rogier P. Voermans

**Affiliations:** ^1^ Department of Gastroenterology and Hepatology Amsterdam University Medical Center Amsterdam the Netherlands; ^2^ Amsterdam Gastroenterology Endocrinology Metabolism Amsterdam the Netherlands; ^3^ Cancer Center Amsterdam Amsterdam the Netherlands

**Keywords:** Editorial, endoscopic ultrasound, Ercp, simulation, training

Advanced endoscopy for pancreatic and biliary diseases has rapidly evolved over the last decades. This exiting evolution is associated with increased complexity and inherent risk of complications. The need for a dedicated training program is evident. However, a structured and consistent program seems to be lacking in Europe. In this issue of *United European Gastroenterology Journal*, Campos et al. describe the results of a survey about current Endoscopic ultrasound (EUS) and Endoscopic retrograde cholangiopancreatography (ERCP) training in Europe.^1^ The aim was to assess the current status of EUS and ERCP training in Europe. The major limitation of this study was the limited number of centers selected and especially the limited number of trainees who responded. Moreover, there is an overrepresentation of known large training hospitals. However, despite these limitations, the authors should be applauded for their effort in conducting this study, since these data can serve as a basis to improve and standardize training program across Europe in the near future.

There are several important outcomes to highlight. First striking finding was that training programs in the 18 included countries differ greatly and are not standardized. For example, only a small number of trainees have structured access to a simulation model and only in about half of the centers a specific curriculum for EUS and ERCP training is available. Also, there is a wide variation regarding the duration of hands‐on training, ranging from one (!) to 24 month(s). Only approximately half of the trainees fulfill European Society of Gastrointestinal Endoscopy (ESGE) guidelines of at least 12 months of high‐volume ERCP training.^2^ Although the ESGE position statement regarding the EUS and ERCP curriculum states that the expected number of procedures for competency are at least 250 diagnostic EUS procedures and at least 300 ERCPs, only 4% of trainees expect to perform at least these numbers. Last but not least, competence assessment varied widely between centers; only about one third used a skills assessment tool such as Direct Observation of Procedural Skills (DOPS) or The EUS and ERCP Skills Assessment Tool (TEESAT).

These results underline the need for a more standardized training. In our opinion, basic prerequisites should include:

1. *Adequate funding for EUS and ERCP fellowships to ensure dedicated training programs*.

In most countries in Europe, post graduate advanced endoscopy fellowship programs for EUS and ERCP are not existing. Training generally takes place during the standard gastroenterology residency for those who are interested in and selected for training in EUS and ERCP. Dedicated time for training is therefore often limited and trainees might benefit from a more structured and formal curriculum to achieve competence. Installing post graduate EUS and ERCP fellowships would lead to more focused training programs; however, funding of such a fellowship hampers implementation in most hospitals and countries.

*2*. *The selection of trainees is based on predefined and transparent criteria, whereas the number of trainees should be limited to the expected number of vacancies.*

When selecting future trainees, apart from applicants motivation and general skills, attracting diverse talent should also be pursued (e.g. gender, ethnicity). Apart from the moral necessity of equity, diversity and inclusion in the workforce are associated with increased creativity, innovation and problem‐solving and should therefore be pursued in each medical subspecialty.^3^, ^4^, ^5^ As to advanced endoscopy, women are highly underrepresented. For example, in advanced endoscopy fellowships in the United States only 14% of fellows were female and 32% of the advanced endoscopy programs never had a female fellow.^6^ The presence of female advanced endoscopy fellows was strongly associated with institutions having female‐advanced endoscopy faculty members. This suggests the importance of female role models to improve gender equality by encouraging female residents to pursue advanced endoscopy.

Furthermore, the expected number of vacancies should be strongly taken into consideration when selecting future trainees. Training too many trainees leads to insufficient exposure and inadequate training for all, thereby declining the training program efficiency. Furthermore, patients are put at unnecessary risks when trainees are trained for high‐risk procedures without eventually proceeding with EUS and ERCP after finalizing their training. Although trainee involvement during an ERCP is not shown to be associated with an overall increased risk of complications or ERCP failure, increased complication risk is associated with suboptimal trainee performance based on the TEESAT score.^7^

3. *Simulation training prior to hands‐on training limit patient risks and optimize the cognitive and technical learning curve.*

Ideally, trainees should start with a central EUS and ERCP course to guarantee appropriate knowledge concerning indications, complications, and materials. Although the benefit in terms of steeper learning curves or fewer complications of simulation‐based training programs for EUS and ERCP is not yet proven, it is likely that it accelerates the technical learning curve. In aviation and surgery simulation models are already a prerequisite before training in daily practice can be commenced and also for colonoscopies the benefit is already proven.^8^ For ERCP this would be especially important since scope handling differs greatly compared to basic gastroscopy and colonoscopy techniques. For EUS, however, simulation‐based training is ideal to improve interpretation of imaging and basic anatomy before hands‐on training. There are mechanical simulators and computer‐based, virtual reality simulators available. Technically, superior computer‐based simulators such as augmented or mixed reality are still in development and not yet ready for implementation.^9^ The duration of the simulation‐based training program and the specific goals to be achieved should be defined in advance. With this step‐up program, junior trainees have not yet taken up space in the workplace; therefore, more hands‐on exposure time will be available for more senior trainees.

4. *Follow a step‐by‐step approach in procedure complexity during hands‐on training.*

To further optimize the learning curve, hands‐on training should preferably start with basic ERCPs before complex ERCPs, that is, start with ERCP Schultz 1/2 before Schultz 3/4 and cannulation post‐sphincterotomy before native papilla. As stated, Voiosu et al. showed that low trainee performance increases the risk of complications for ERCP. High‐risk procedures with low performance trainee involvement showed a remarkably high complication or technical failure rate of 36.6%.^7^ Trainees should achieve a predefined competence level based on DOPS or TEEAT before participating in high‐complex procedures. To achieve this step‐by‐step approach in procedure complexity, trainees should preferably start their training in a hospital that has a substantial number of routine ERCPs and then continue training in a tertiary/academic center for more advanced EUS and ERCP. Finally, training in therapeutic EUS should only be considered once competence in ERCP as well as diagnostic EUS (including EUS guided fine needle biopsy/aspiration) is achieved. Preferably in post‐graduate fellowships.

5. *Minimum 12 months exposure time, preferably uninterrupted. Extent, shorten, or stop training the specific trainee based on competence DOPS and/or TEESAT.*

Traditionally, the apprenticeship model is the basis of (advanced) endoscopic training, expanding trainee actions step‐by‐step in real patients under direct supervision. In this model, competence is determined based on a number of procedures performed. The limitations of this volume‐based assessment of competence is widely acknowledged and has led to a shift from a volume‐based to an outcomes‐based approach, focusing on predefines competency outcomes.^9^, ^10^, ^11^ Observational assessment tools can be used to evaluate trainee's progression and competence, containing a mix of technical, cognitive and integrative skills, but also pre‐ and post‐procedural, and specific procedure‐related outcomes such as cannulation rate, complication rate, and yield of fine needle aspiration/biopsy (FNA/B). The incorporation of formal observational assessment tools such as DOPS or TEESAT in a training program is recommended by the ESGE; both these assessment tools have shown strong evidence of validity for ERCP.^12^ Occurrence of complications should also be assessed to evaluate competence. On average, competence in diagnostic EUS is achieved in 250 cases and for ERCP after 300 cases.^13^, ^14^ However, by adapting the number needed based on competences instead of volume, some trainees will be qualified with fewer numbers and some will need an extension to achieve the competence threshold. This will hopefully be in balance so adequate volume for all trainees will be achieved.

Ideally, training for advanced endoscopy will take place for at least 12 months without interruption. Since starting and/or expanding a family generally takes place during medical residency or post graduate training, pregnancy, lactation and paternity/maternity leave will often lead to curricular interruptions. These interruptions should not be considered a burden; consequently, extension of exposure time should be possible to create an inclusive environment.

The development and implementation of a standardized EUS and ERCP training program in Europe is crucial. The study of *Campos et al.* underlines that current programs vary widely and that basic prerequisites for adequate training are lacking in most centers/countries. The results of this study should serve as a basis to optimize and standardize EUS and ERCP training. Requirements necessary for a dedicated curriculum, preferably in the setting of a dedicated post graduate fellowship, are listed above. Facilities such as a central training course and simulation‐based training should ideally be centralized in Europe. Although each country is responsible for its own curriculum and trainees, teaching hospitals across Europe should collaborate to guarantee high‐quality uniform training programs to ensure competence before independent practice.

## CONFLICT OF INTEREST STATEMENT

Selma J. Lekkerkerker none. Rogier P. Voermans reports unrestricted research grant from Boston Scientific and Prion medical and speakers fees from Zambon and Viatris.

## Data Availability

Data sharing is not applicable to this article as no new data were created or analyzed in this study.
